# Long-Term Exposure of Psoralen and Isopsoralen Induced Hepatotoxicity and Serum Metabolites Profiles Changes in Female Rats

**DOI:** 10.3390/metabo9110263

**Published:** 2019-11-02

**Authors:** Yingli Yu, Pengli Wang, Ruili Yu, Jiaxi Lu, Miaomiao Jiang, Kun Zhou

**Affiliations:** 1 Institute of Traditional Chinese Medicine, Tianjin University of Traditional Chinese Medicine, Tianjin 300193, China; yuguyu88@sina.com (Y.Y.);; 2 Tianjin Key Laboratory of Chinese medicine Pharmacology, Tianjin 300193, China; 3 Tianjin State Key Laboratory of Modern Chinese Medicine, Tianjin 300193, China

**Keywords:** *Fructus Psoraleae*, psoralen, isopsoralen, sd rats, toxicity, metabolomics

## Abstract

Pre-clinical safety evaluation of traditional medicines is imperative because of the universality of drug-induced adverse reactions. Psoralen and isopsoralen are the major active molecules and quality-control components of a traditional herbal medicine which is popularly used in Asia, *Fructus Psoraleae*. The purpose of this study is to assess the long-term effects of psoralen and isopsoralen with low levels on the biochemical parameters and metabolic profiles of rats. Three doses (14, 28, and 56 mg/kg) of psoralen and one dose (28 mg/kg) of isopsoralen were administered to rats over 12 weeks. Blood and selected tissue samples were collected and analyzed for hematology, serum biochemistry, and histopathology. Metabolic changes in serum samples were detected via proton nuclear magnetic resonance (^1^H-NMR) spectroscopy. We found that psoralen significantly changed the visceral coefficients, blood biochemical parameters, and histopathology, and isopsoralen extra influenced the hematological index. Moreover, psoralen induced remarkable elevations of forvaline, isoleucine, isobutyrate, alanine, acetone, pyruvate, glutamine, citrate, unsaturated lipids, choline, creatine, phenylalanine, and 4-hydroxybenzoate, and significant reductions of ethanol and dimethyl sulfone. Isopsoralen only induced a few remarkable changes of metabolites. These results suggest that chronic exposure to low-level of psoralen causes a disturbance in alanine metabolism, glutamate metabolism, urea cycle, glucose-alanine cycle, ammonia recycling, glycine, and serine metabolism pathways. Psoralen and isopsoralen showed different toxicity characteristics to the rats.

## 1. Introduction

*Fructus Psoraleae* (F.P.), the seed of *Psoralea corylifolia* L. (Leguminosae), has been widely used in Asian countries as a traditional medicine with the effects of warming kidney [1], activating “yang,” promoting inspiration [2], and checking diarrhea [3]. There are more than 40 prescriptions containing F.P. in the Chinese pharmacopoeia. However, with the continuously expanded clinical application of F.P., toxicity problems have been concerned by researchers and doctors. Yao et al. [4] claimed that F.P. is one of the emerging hepatotoxins. Oral administration of the F.P. aqueous extract (25 g/kg) induced significantly liver and kidney injuries in Wistar rats, and the biomarkers related to glycerol phospholipids metabolism, amino acid metabolism, and energy metabolism, were largely changed [5]. Psoralen (P) and isopsoralen (IP) are the major active molecules and quality-control components of F.P. [6]. Many investigations have been conducted on the pharmacological properties of P and IP, such as antibacterial [7], anti- inflammatory [8], antitumor [9], estrogen-like effect, and antioxidant activities [10]. However, regardless of their therapeutic effects, high doses of P or IP are proved to be poisonous [11,12] and made major contribution to the toxicity induced by F.P. A study (in Chinese) [13] about the toxicity intensity of F.P. prepared by different processing methods suggested that there is a positive correlation between the toxicity and content of P and IP. However, there lacks a systematic study of the toxicity, especially the long-term toxicity of P and IP. Few studies have been conducted on the mechanism of P or IP or their comparison.

The traditional toxicity assessment method using biochemistry and histopathology lacks sensitivity and accuracy. Metabonomics [14] is a relatively new research technique and has become a viable tool for investigating the biochemical effects of many toxic substances. It reflects the overall changes of the body through the metabolic changes of plasma or urine, during a genetic modification or physiological stimulus [15]. With the continuous progress of the discipline, untargeted metabonomics has been widely used in drug safety assessment and toxicity prediction and provided valuable information for drug-induced cardiotoxicity, hepatotoxicity, and nephrotoxicity.

In this study, the long-term toxicities induced by psoralen (P) and isopsoralen (IP) were performed in SD rats with a 12-week oral administration of P (14, 28, 56 mg/kg) or IP (28 mg/kg), and the reversibility, persistence, or delayed occurrence of target organ toxicity were detected after a 4-week recovery period. The results illustrated that chronic exposure to low-level of psoralen or isopsoralen injured the visceral coefficients, hematological and blood biochemical parameters, and histopathology of rats. Besides, psoralen caused a disturbance in alanine metabolism, glutamate metabolism, urea cycle, glucose-alanine cycle, ammonia recycling, glycine and serine metabolism pathways. Psoralen and isopsoralen showed different toxicity characteristics to the rats.

## 2. Results

### 2.1. Effects on the General Behavior and Mortality

The administration of psoralen or isopsoralen for 12 weeks did not induce any behavioral modification at any time point in treated rats compared to their respective control group. The color, appearance, and behavior of the rats were normal and no death was recorded during the experimental period.

### 2.2. Effects on the Body Weight

**Figure 1:**
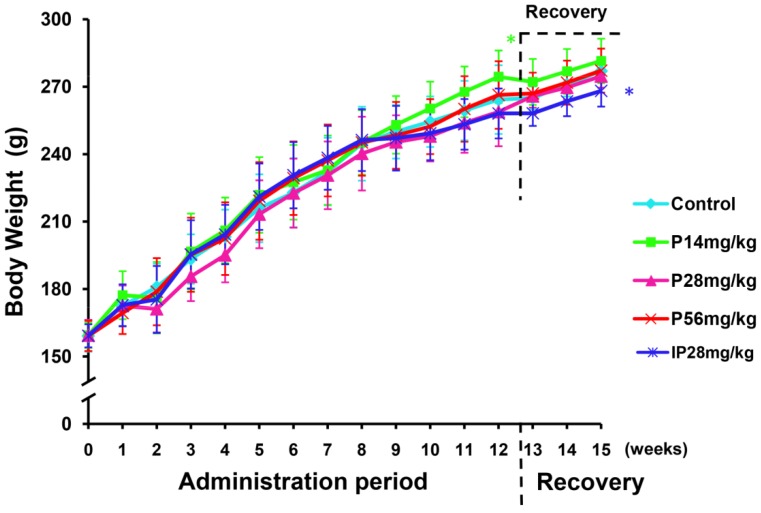
Weights of rats during 3 months of psoralen or isopsoralen administration and recovery period. Data represented as mean ± SD. * P < 0.05, significant difference from control rats.

Body weight of each animal was measured every week throughout the study. Figure 1 shows a graphical representation of average weights of rats during psoralen or isopsoralen administration and recovery periods. In all groups, body weights gradually increased from the 1st week to the 16th week, and body weights of animals in the treatment groups showed no significant decrease compared with that of control during 12 weeks of drug administration. However, the weights of the isopsoralen group were significantly lower than those of the control group (Figure 1, *P* < 0.05) at the end of recovery period.

### 2.3. Effects on the Food and Water Intakes

**Figure 2:**
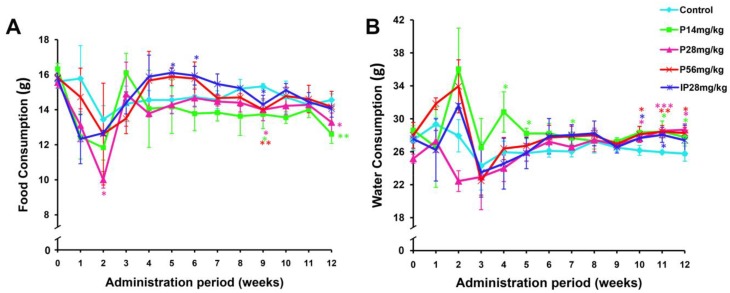
The rats’ food and water intake within 3 months’ repeated administration of psoralen or isopsoralen. (**A**) Food consumption. (**B**) Water consumption. Data represented as mean ± SD. * *P* < 0.05, ** *P* < 0.01, *** *P* < 0.001, significant difference from control rats.

Figure 2A,B represent the effects of psoralen or isopsoralen on the food and water intakes in sub-acute treatment. It was noted that there was no consistent effects of food intake or water consumption by repeated oral administration of psoralen (14, 28 and 56 mg/kg) or isopsoralen (28 mg/kg) for SD rats in comparison with the control in the first 8 weeks. However, in the 9th week, the food intakes in the treated groups were significantly lower than those in the control group (Figure 2A, *P* < 0.05 or *P* < 0.01). And the water consumptions in the psoralen (14, 28, and 56 mg/kg) and isopsoralen groups were significantly higher than those in the control group (Figure 2B, *P* < 0.05, *P* < 0.01, or *P* < 0.001) from the 9th week to the end.

### 2.4. Effects on the Visceral Coefficients

Organ-to-body weight ratio (relative organ weight) is a sensitive index for toxicity evaluation. The visceral coefficients of rats after 3 months of repeated administration and 2 weeks of recovery are shown in Table 1. Compared with the control group, the spleen coefficients in all groups treated with psoralen (14, 28, and 56 mg/kg) increased significantly (*P* < 0.05 or *P* < 0.01), and the thymus coefficients in the middle-dose group (28 mg/kg) and high-dose group (56 mg/kg) decreased significantly (all *P* < 0.01). Besides, the heart and adrenal gland coefficients significantly increased (all *P* < 0.05), and the liver coefficient significantly decreased (*P* < 0.01) in the psoralen low-dose group (14 mg/kg). In the psoralen middle-dose group (28 mg/kg), the brain coefficient increased significantly (*P* < 0.05), while the liver coefficient significantly decreased (*P* < 0.05). Moreover, daily administration of isopsoralen (28 mg/kg) significantly increased the coefficients of brain and heart (*P* < 0.05 and *P* < 0.001). However, in the recovery period, only a significant increase (*P* < 0.05) in liver coefficient of the psoralen low-dose group (14 mg/kg) and a decrease (*P* < 0.05) in ovary coefficient of the psoralen high-dose group (56 mg/kg) were observed. No statistically significant difference was detected in other visceral coefficients.

### 2.5. Effects on Hematological Parameters

The hematopoietic system is one of the most sensitive targets for toxic chemicals and served as an important index of overall health status for both humans and animals [16]. The hematological parameters of the experimental and control rats are presented in Table 2. There were no significant changes observed in the psoralen middle-dose (28 mg/kg) and high-dose (56 mg/kg) groups when compared with the control group (*P >* 0.05), although a significant change was found in white blood cell (WBC) in the psoralen low-dose (14 mg/kg) group. More importantly, in the isopsoralen group (28 mg/kg), there were significant increase in the percentage of white blood cells (WBC) (*P* < 0.05) and decreases in hemoglobin (HGB), mean corpuscular HGB (MCH), and mean corpuscular HGB concentration (MCHC) (all *P* < 0.001). After the recovery period, all the changed parameters in the isopsoralen group were returned to the range of normal physiological variations compared to the control (*P >* 0.05).

### 2.6. Effects on Blood Biochemical Parameters

The effects of repeated administration of psoralen or isopsoralen and recovery in rats on serum biochemical parameters are summarized in Table 3. All doses of psoralen (14, 28 and 56 mg/kg) and isopsoralen (28 mg/kg) treatment significantly decreased the relative levels of ALP, ALB, BUN, CK, and GLU (Table 3, *P* < 0.05, *P* < 0.01 or *P* < 0.001) in comparison with the control group. Furthermore, low-dose of psoralen (14 mg/kg) reduced the TG level at 12th-week administration (*P* < 0.05). No statistically significant differences were observed among the levels of ALT, AST, TBIL, TC, TP, or CRE between the control and treated animals (*P >* 0.05). However, in the recovery period, all the damage was abolished except for increases of AST in the psoralen low-dose (14 mg/kg) group (*P* < 0.05) and BUN in the psoralen low-dose (14 mg/kg) and middle-dose (28 mg/kg) groups (*P* < 0.01 and *P* < 0.05, respectively).

### 2.7. Effects on the Histopathology of the Major Organs

**Figure 3:**
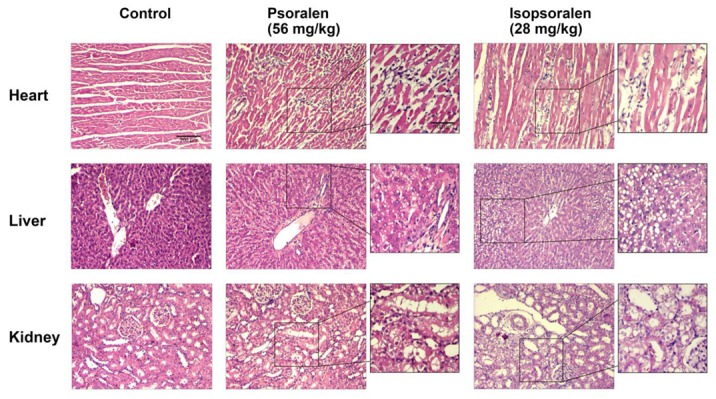
Histopathological photographs of organ lesions in Sprague–Dawley (SD) rats. H & E staining showing the heart, liver, and kidney injuries induced by psoralen and isopsoralen, 100× and 200×.

Histopathological examination of the major organs is presented in Figure 3. Some individual pathological changes were observed in the psoralen high-dose (56 mg/kg) group and isopsoralen (28 mg/kg) group. The major pathological findings from the histopathological examination included portal myocardial inflammatory cell infiltration in the heart, minimal inflammatory cell foci, and vacuolar degeneration in the liver, and distal convoluted tubule cortical vacuolar degeneration and protein cast in the kidneys (Figure 3). There was no obvious pathological change in the animals of recovery period.

### 2.8. Metabolites Present in Serum Samples

**Figure 4:**
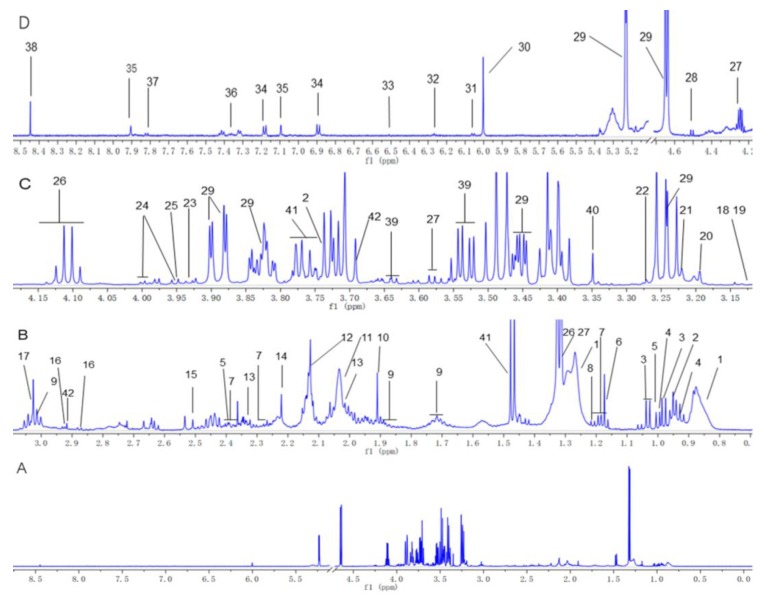
1H-NMR spectra of rat plasma sample. (**A**) Full 1H-NMR spectrum from δ 0.0 to 9.0. (**B**) Enlarged spectrum from δ 0.7 to 3.1. (**C**) Enlarged spectrum from δ 3.1 to 4.17. (**D**) Enlarged spectrum from δ 4.2–8.5. Peaks of 42 metabolites: 1, lipids; 2, leucine; 3,valine; 4, isoleucine; 5, isobutyrate; 6, ethanol; 7, 3-hydroxybutyrate; 8, methylmalonate; 9, lysine; 10, acetate; 11, N-acetyl groups; 12, *O*-acetyl groups; 13, proline; 14, acetone; 15, citrate; 16,asparagine; 17, ornithine;18, citrulline; 19, malonate; 20, choline; 21, trimethylamine oxide; 22, glycerophosphocholine; 23, creatine; 24, serine; 25, creatine phosphate; 26, lactate; 27, threonine; 28, arabinose; 29, glucose; 30, maleate; 31, adenosine; 32, thymidine; 33, fumarate; 34, tyrosine; 35, histidine; 36, phenylalanine; 37, 4-hydroxybenzoate; 38, formate; 39, glycerol; 40, methanol; 41, alanine; 42, dimethylglycine.

Typical ^1^H-NMR spectrum of rat serum sample is shown in Figure 4 and the proton signals were assigned to 42 metabolites according to the literatures, standard references, public databases, and information from two-dimensional NMR spectra. These metabolites included amino acids (alanine, asparagine, glutamine, isoleucine, leucine, valine, lysine, glycine, histidine, phenylalanine, proline, serine, tyrosine, methionine, threonine, ornithine and citrulline), saturated and unsaturated lipids, ketone bodies (acetone and 3-hydroxybutyrate), carbohydrates (glucose, arabinose), TCA cycle intermediates (citrate and fumarate), nucleic acids (adenosine and thymidine), some other organic acids (2-phenylpropionate, 4-hydroxybenzoate, pyruvate, lactate, acetate, isobutyrate, maleate, malonate, methylmalonate and formate), dimethyl sulfone, ethanol, glycerol, methanol, *N*-acetyl, and *O*-acetyl glycoprotein together with choline metabolites including choline, phosphorylcholine, glycerophosphorylcholine, dimethylamine, dimethylglycine, trimethylamine oxide, creatine, and creatine phosphate.

### 2.9. Screening for Differential Metabolites

**Figure 5:**
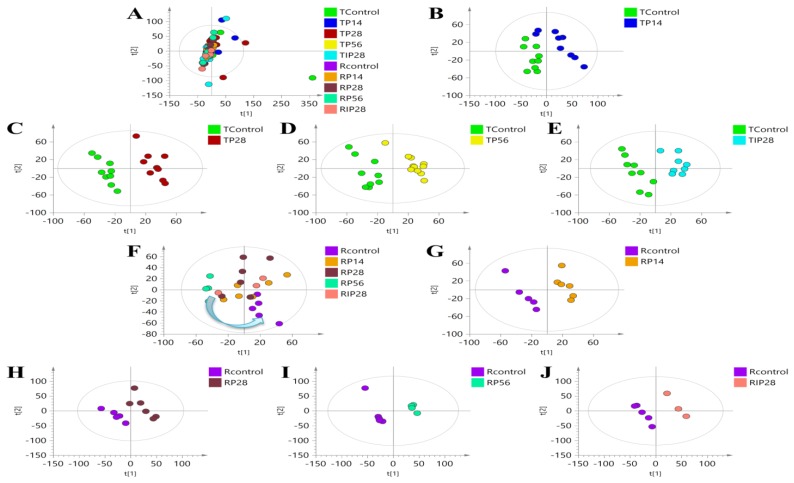
Comparative analyses of metabolic profiles in serum samples among different groups. (**A**) PCA scores plot of all the groups; (**B**) PLS-DA scores plot between the control and low-dose psoralen (14 mg/kg) groups; (**C**) PLS-DA scores plot between the control and medium-dose psoralen (28 mg/kg) groups; (**D**) PLS-DA scores plot between the control and high-dose psoralen (56 mg/kg) groups; (**E**) PLS-DA scores plot between the control and medium-dose isopsoralen (28 mg/kg) groups; (**F**) PLS-DA scores plot among the control and drug-treated groups in recovery period; (**G**) PLS-DA scores plot between the control and low-dose psoralen (14 mg/kg) groups in recovery period; (**H**) PLS-DA scores plot between the control and medium-dose psoralen (28 mg/kg) groups in recovery period; (**I**) PLS-DA scores plot between the control and high-dose psoralen (56 mg/kg) groups in recovery period; (**J**) PLS-DA scores plot between the control and medium-dose isopsoralen (28 mg/kg) groups in recovery period.

The binning NMR data were normalized and subjected to non-supervised principle components analysis (PCA) models. The scores plot of PCA (Figure 5A) showed a few outliers out of 95% confidence intervals had to be removed. To extensively investigate metabolic alterations that associated with psoralen and isopsoralen toxicities, we applied supervised partial least squares discriminate analysis (PLS-DA) to distinct animals dosed with drugs and their corresponding controls. PLS-DA models between psoralen groups and controls in administration period were found to be valid with CV-ANOVA (*P* < 0.05) and permutation test (permutation number = 200), whereas all the rest PLS-DA models in pair wise comparisons had unacceptable *P* values of CV-ANOVA, R^2^, or Q^2^ values (Table 4).

**Figure 6:**
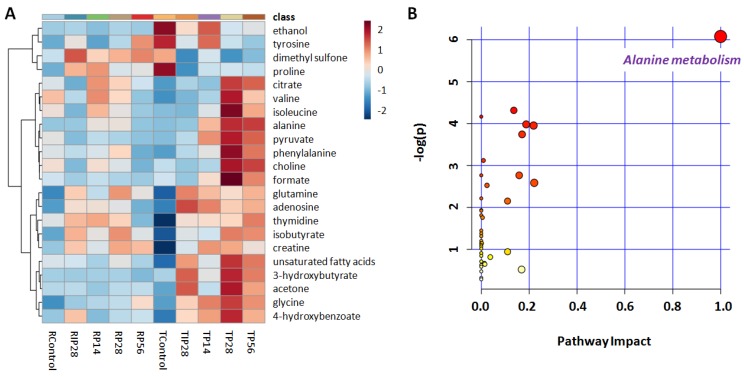
(**A**) Heat maps for average contents of 22 differential metabolites in all the groups with normalized data and auto-scale features. (**B**) Bubble plot of pathway enrichment analysis of 22 differential metabolites induced by high-dose psoralen (56 mg/kg).

Metabolites that contributed most to prediction of the class were selected by VIP and false discovery rate (FDR) adjusted *P* values of t-test. Compared with controls, both high dosage (56 mg/kg) and medium dosage (28 mg/kg) of psoralen induced remarkable elevations of forvaline, isoleucine, isobutyrate, alanine, acetone, pyruvate, glutamine, citrate, unsaturated lipids, choline, creatine, phenylalanine, and 4-hydroxybenzoate, and significant reductions of ethanol and dimethyl sulfone. High-dose (56 mg/kg) of psoralen extra induced significant increases of 3-hydroxybutyrate, glycine, adenosine, thymidine, and formate and decreases of proline and tyrosine. No significant metabolic differences were observable between the low-dose psoralen (14 mg/kg) group and controls. Isopsoralen (28 mg/kg) only induced a few remarkable changes of metabolites, including glutamine elevation and decreases for ethanol and dimethyl sulfone (Figure 6).

At 4 weeks post dose, the metabolomic recovery of all the treated groups judged from the rigorously tested PLS-DA models and T-tests in terms of the number of changed metabolites and degree of changes (Table 5 and Figure 5). No significant differences of potential metabolites related with psoralen and isopsoralen injury were observed compared with controls at the end of the recovery time point (Table 6).

## 3. Discussion

**Figure 7:**
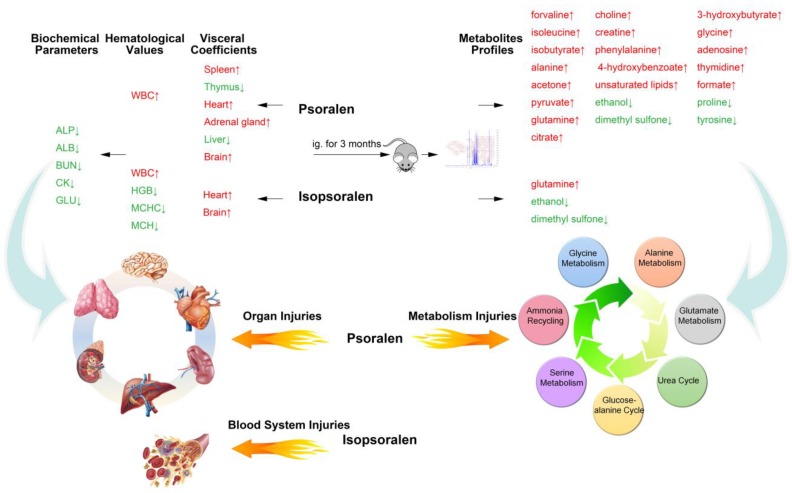
Schematic diagram illustrating the hepatotoxic effect of psoralen and isopsoralen on rats.

*Fructus Psoraleae* (F.P.), commonly known as “Bu-Gu-Zhi” in China, have been used in traditional medicine to treat diseases such as leukoderma [17], osteoporosis [18], bacterial infection [3], and impotence and have been demonstrated to have antitumor effects [9]. However, with the continuously expanded clinical application of F.P., the concerns have risen about the safe medication of F.P. and the toxicity induced has attracted increasing attention. A study [19] summarized the clinical reports related to the drug safety of F.P. from 1978 to 2016 in China, and found that the adverse reactions involved in F.P. include liver damage, acute aplastic anemia after drug-induced sub-acute severe hepatitis, and phototoxic contact dermatitis. Wang et al. [20] collected a total of 42 hepatic injury cases in 26 literatures, which involved 11 kinds of Chinese patent medicine containing F.P. Toxicology studies on animals [5] also suggest that F.P. mainly exhibits toxicity toward the liver. But no related studies have systematically focused on the toxicity and metabolic influence of the main active compounds, psoralen and isopsoralen. The present study assessed the toxic effects and the metabolomics influence of 12-week repeated oral administration of psoralen and isopsoralen in SD rats at different dose levels, and the reversibility, persistence or delayed occurrence of target organ toxicity were detected after a 4-week recovery period. Our results indicated that long-term exposure to low levels of psoralen or isopsoralen could induce serious toxic reaction. Moreover, psoralen showed more injuries on the visceral organs than isopsoralen, while isopsoralen damaged the blood system more seriously (Figure 7). Besides, psoralen induced a series of alterations in alanine metabolism, glutamate metabolism, urea cycle, glucose-alanine cycle, ammonia recycling, glycine and serine metabolism pathways. However, the toxicity induced both by psoralen and isopsoralen are reversible, which are abolished after a period of recovery.

Although the body weights were not deviant changed in the treated rats compared to their respective control group, the significantly decreased food intake and increased water consumption indicated the impaired physical condition, both in the psoralen (14, 28, and 56 mg/kg) groups and in the isopsoralen (28 mg/kg) group. Furthermore, the abnormal increase in visceral coefficient is an indication of cell swelling or inflammation, while a reduction can be attributed to cellular constriction [21]. Compared with the control group, psoralen significantly increased the spleen coefficients (14, 28 and 56 mg/kg, *P* < 0.05 or *P* < 0.01), brain coefficient (28 mg/kg, *P* < 0.05), the heart and adrenal gland coefficients (14 mg/kg, all *P* < 0.05), decreased the thymus coefficients (28 and 56 mg/kg, *P* < 0.01), and the liver coefficient (14 and 28 mg/kg, *P* < 0.01). While isopsoralen (28 mg/kg) only increased the coefficients of brain and heart (*P* < 0.05 and *P* < 0.001). Consistently, histopathological examination found markedly pathological changes in the heart, liver, and kidneys in the psoralen- and isopsoralen-treated groups. These results suggested that psoralen injured the visceral organs more seriously than isopsoralen, and maybe make more contribution to the liver injury induced by *Fructus Psoraleae*. In the previous studies about the acute hepatotoxicity of psoralen and isopsoralen [22], high dosage are usually used and the toxic reactions occurred quickly and severely, which probably mask the underlying differences of the two ingredients that induced toxic reactions. Our findings imply that the chronic and cumulative toxicity caused by psoralen or isopsoralen can be delayed [23] for 3 months to be appear after administration. And psoralen may have more toxic effects on the spleen, adrenal gland, thymus, and liver than isopsoralen at the same dose, which are all reversible with a period of recovery. In the recovery period, most of the organ changes were abolished, with only a significant increase (*P* < 0.05) in liver coefficient of the psoralen low-dose group (14 mg/kg) and a decrease (*P* < 0.05) in ovary coefficient of the psoralen high-dose group (56 mg/kg) observed.

With respect to the hematology, psoralen and isopsoralen treatment induced dysfunctions accompanied by changes of the various parameters. In our study, a significant change was found in white blood cell (WBC) in the psoralen (14 mg/kg) group. More importantly, in the isopsoralen (28 mg/kg) group, there were significant increase in the percentage of white blood cells (WBC) (*P* < 0.05) and decreases in hemoglobin(HGB), mean corpuscular HGB (MCH), and mean corpuscular HGB concentration (MCHC) (all *P* < 0.001). It was reported that *Fructus Psoraleae* induced acute aplastic anemia after administration, and our results implied that isopsoralen is more likely related to the inflammation and anemia induced by Fructus Psoraleae. While after the recovery period, all the changed parameters in the isopsoralen group were returned to the range of normal physiological variation as compared to the control (*P >* 0.05).

The serum ALT, AST, and ALP activities are commonly used as markers of liver damage [11]. Our results indicated that all doses of psoralen (14, 28, and 56 mg/kg) and isopsoralen (28 mg/kg) treatment significantly decreased the relative levels of ALP, ALB, BUN, CK, and GLU (Table 3, *P* < 0.05, *P* < 0.01 or *P* < 0.001) in comparison with the control group. The changed hepatic parameters suggested that the oral administration of psoralen and isopsoralen negatively affected the livers of the animals, which can be detected under low concentration exposure. Recently, studies about toxicity assessment [24] claimed that some pollutants, drugs, and natural substances do not follow the rule that high dose tests exhibit healthy problems which are found in low dose exposure. In our study, low-dose of psoralen (14 mg/kg) reduced the TG level at the 12th-week administration (*P* < 0.05), but middle-dose and high-dose did not. The results inspired us that the long-term exposure might induce accumulated psoralen in the body and injure metabolic enzymes. We have previously found that psoralen and isopsoralen injured the liver through dislocating the cytochrome P450 metabolism [25], which maybe the main targets of psoralen and isopsoralen in the body. However, in the recovery period, all the damage were abolished except for the increases of AST (14 mg/kg, *P* < 0.05) and BUN (14 and 28 mg/kg, *P* < 0.01 and *P* < 0.05, respectively).

However traditional experience is not effective at identifying herbs or herb combinations that cause cumulative, chronic, or delayed toxicity [26]. Metabolic profiling analysis revealed that psoralen dose-dependently and reversibly induced a series of alterations in metabolisms in serum samples of female SD rats, mainly enriching into alanine metabolism, glutamate metabolism, urea cycle, glucose-alanine cycle, ammonia recycling, glycine, and serine metabolism pathways with their *P* values less than 0.05. Among them, only the impact factor of alanine metabolism was nearly to 1 and much higher than the others, indicating its importance to reflect psoralen impact. Three metabolites (alanine, pyruvate and glycine) hit on the pathway and all their concentrations in serum showed significant elevations after psoralen intake. The increase of alanine was consistent with psoralen-induced activity enhancement of alanine transaminase in rat liver. The enzyme can catalyze transamination reaction by the reductive amination of pyruvate to produce alanine. Some other amino acids, such as valine, isoleucine, glutamine, phenylalanine, were also observed with level increases in psoralen-treated group, implying psoralen probably inhibited free amino acids to synthesize protein as well as to prevent them be substrates from entering gluconeogenesis in rats. This study provides the thoughts that an analysis of the metabolic profiles can contribute to the understanding of the adverse effects of long-term exposure to the potentially toxic traditional Chinese medicine.

## 4. Materials and Methods

### 4.1. Animals and Long-Term Toxicity Treatment

Psoralen and isopsoralen were purchased from Tianjin Crescent Lake Biotech Co. Ltd. (Tianjin, China). Adult female Sprague–Dawley (SD) rats, aged 4 weeks and weighing 85–100 g were obtained from the specific pathogen-free facility of Beijing HFK Bioscience Technology Co. Ltd. (Beijing, China). The animals were transferred to laboratory and submitted to adaptation by a period of 7 days. They were acclimatized under controlled temperature (25 ± 2 °C) and humidity (50–70%) on a 12-h light/12-h dark cycle (artificial lighting from 08:00 to 20:00), and had free access to standard chow and drinking water. The protocols of the animal experiments were approved by the Laboratory Animal Ethics Committee of Tianjin University of Traditional Chinese Medicine (permit number: TCM-LAEC 2016001).

A total of 90 rats were randomly divided into five groups with 18 rats in each group: (1) Control group (Tcontrol); (2) high-dose (56 mg/kg) of psoralen treated group (TP56); (3) medium-dose (28 mg/kg) of psoralen treated group (TP28); (4) low-dose (14mg/kg) of psoralen treated group (TP14); and (5) a dose of 28 mg/kg isopsoralen treated group (TIP28). The rats in the P and IP groups were treated with drugs at the specified doses by intragastric administration once a day for 3 months, and the rats in the control group were treated with gum tragacanth solution (drugs solvent) only. The general behavior of the rats were observed and recorded daily. The weight, food consumption, and water consumption of the rats were detected weekly.

After 3 months of drug administration, 6 animals per group entered a 4-week recovery period to observe reversibility, persistence or delayed occurrence of toxic effects. Then the group names for short were changed into RControl, RP56, RP28, RP14 and RIP28, respectively.

### 4.2. Organ Weights, Gross Necropsy and Histopathological Examination

At the end of the administration and recovery periods, animals were fasted overnight, but with water ad libitum. The rats were anesthetized with 10% chloral hydrate (Shanghai Reagent Co., td, Shanghai, China) 500 µL/100 g by intraperitoneal injection, and sacrificed by decapitation after blood collection from the abdominalaorta. Blood was collected to examine serum biochemical parameters, and the following organs were carefully dissected out and weighed after dissection to remove fat and connective tissue: brain, heart, liver, thymus glands, spleen, kidneys, adrenal glands, ovary, and uterus. The ratio of each organ to terminal body weight (relative organ weight) was calculated respectively. At the end of the recovery period, the remaining rats were treated by the same method.

The major organs/tissues of all the animals were fixed in 10% neutral buffer formalin at the end of the administration period. Histopathological examination was performed on the 56 mg/kg of P, 28 mg/kg of IP administrated groups, and the control group, during both treatment and recovery periods. The fixed organs were paraffin-embedded, microsected into nominal thickness of 5 μm, stained with hematoxylin and eosin, and then recorded under optical microscopy (OLYMPUSBX51, OLYMPUS CO., Tokyo, Japan).

### 4.3. Hematological and Biochemical Parameters

At the end of the administration on week 12 and recovery periods on week 16, blood samples were obtained for analysis of hematology and serum biochemistry. All rats were fasting but allowed access to water ad libitum overnight prior to blood sample collection. The blood sample of each rat was collected into two tubes, the heparinized centrifuge tube and dry non-heparinized centrifuge tube. The heparinized blood including white blood cell (WBC), red blood cell (RBC), hemoglobin (HGB), mean corpuscular volume (MCV), mean corpuscular HGB (MCH), mean corpuscular HGB concentration (MCHC), and platelets (PLT) was used for a hematological determination according to the manufacturer’s operator manual. Plasma was isolated and used to determine prothrombin time (PT). For serum biochemical analysis, the non-heparinized blood was centrifuged at 3500 rpm (10 min, 4 °C) and the supernatants were collected. Several serum biochemical parameters were detected by an automatic blood biochemical analyzer (HITACHI 7020; Hitachi, Tokyo, Japan), which measured alanine amino transferase activity(ALT), aspartate amino transferase activity (AST), alkaline phosphatase activity (ALP), total bilirubin (TBIL), total cholesterol (TC), triglycerides(TG), total protein (TP), albumin (ALB), blood urea nitrogen (BUN), creatinine (CRE), creatine kinase (CK), and glucose (GLU). All parameters of blood chemistry and hematology were measured following standard procedures.

### 4.4. Sample Preparation and NMR Measurements

A volume of 300 μL serum samples were added with 200 μL of phosphate buffer in D_2_O (0.5 M, pD = 7.4) and transferred into 5 mm NMR tubes (Norell, USA) for analysis. The NMR spectra were acquired at 300 K on a 600.13 MHz Bruker AVIII HD spectrometer (Bruker BioSpin, Germany) equipped with a 5 mm BBO H&F cryogenic probe. Automatic shimming and adjusting of 90° pulse length were performed for each sample. Standard Carr-Purcell-Meiboom-Gill (CPMG) pulse sequence was used to suppress the residual water signal and filter macromolecular signals. A total of 64 scans were collected into 32K data points over a spectral width of 12019.2Hz with a relaxation delay of 4 s and an acquisition time of 3.07 s.

### 4.5. Data Processing for Metabonomic Analysis

Proton NMR data were processed with MestNova 6.1.1 (Mestrelab Research S.L., Compostela, Spain). Spectra were carefully phased, baseline corrected and referenced to the aldehyde proton signal of formate (8.444 ppm). The spectral region from *δ* 0.5 to 9.0 was integrated into bins with an equal width of 0.001 ppm and residual solvent signal of water from *δ* 4.73 to 5.85 was discarded during analysis.

Multivariate statistical analysis was conducted using the web server for metabonomic data analysis: Metaboanalyst 3.0 (www.metaboanalyst.ca). Partial least squares discriminate analysis (PLS-DA) was carried out on normalized NMR data for group clustering and biomarker identification. The distinct metabolites as potential markers between classes were weighted through the values of variable importance in the projection (VIP > 1), fold change (FC > 1.5), and Student T-test (*P* < 0.05).

### 4.6. Statistical Analysis

The data were formulated as mean ± S.D. for the tables and mean ±S.E.M for the figures. The significance of differences between groups was evaluated by one-way analysis of variance (ANOVA) and Dunnett’s *t*-test. Differences were considered statistically significant at *P* < 0.05.

## Figures and Tables

**Table 1:** Visceral coefficients of rats after 3 months of repeated administration and 4 weeks of recovery, respectively (mean ± SD, *n* = 12 for administration and *n* = 6 for recovery).

Organs	Dose	Brain	Heart	Liver	Thymus	Spleen	Kidney	Adrenal Gland	Ovary	Uterus
12 weeks	Control	0.648 ± 0.064	0.295 ± 0.023	3.000 ± 0.202	0.150 ± 0.025	0.180 ± 0.020	0.675 ± 0.062	0.026 ± 0.005	0.063 ± 0.011	0.175 ± 0.036
	P14 mg/kg	0.687 ± 0.044	0.320 ± 0.028 *	2.653 ± 0.207 **	0.140 ± 0.026	0.216 ± 0.041 *	0.673 ± 0.040	0.031 ± 0.003 *	0.066 ± 0.007	0.155 ± 0.028
	P28 mg/kg	0.710 ± 0.050 *	0.325 ± 0.030	2.731 ± 0.318 *	0.120 ± 0.028 **	0.211 ± 0.027 **	0.714 ± 0.060	0.029 ± 0.006	0.070 ± 0.015	0.160 ± 0.036
	P56 mg/kg	0.685 ± 0.046	0.307 ± 0.021	2.876 ± 0.181	0.121 ± 0.026 **	0.230 ± 0.040 **	0.672 ± 0.029	0.027 ± 0.005	0.064 ± 0.010	0.183 ± 0.068
	IP28 mg/kg	0.696 ± 0.045 *	0.348 ± 0.028 ***	2.793 ± 0.368	0.149 ± 0.046	0.209 ± 0.051	0.696 ± 0.043	0.028 ± 0.006	0.065 ± 0.009	0.153 ± 0.031
Recovery	Control	0.575 ± 0.040	0.335 ± 0.020	2.584 ± 0.230	0.168 ± 0.018	0.180 ± 0.021	0.603 ± 0.046	0.021 ± 0.005	0.050 ± 0.004	0.192 ± 0.075
	P14 mg/kg	0.590 ± 0.043	0.355 ± 0.020	2.894 ± 0.131 *	0.157 ± 0.016	0.178 ± 0.021	0.557 ± 0.153	0.021 ± 0.004	0.048 ± 0.009	0.162 ± 0.022
	P28 mg/kg	0.583 ± 0.077	0.354 ± 0.020	2.781 ± 0.137	0.182 ± 0.031	0.192 ± 0.020	0.618 ± 0.029	0.020 ± 0.004	0.045 ± 0.007	0.147 ± 0.015
	P56 mg/kg	0.567 ± 0.034	0.334 ± 0.015	2.592 ± 0.138	0.153 ± 0.021	0.186 ± 0.014	0.624 ± 0.038	0.023 ± 0.002	0.044 ± 0.003 *	0.227 ± 0.111
	IP28 mg/kg	0.605 ± 0.048	0.350 ± 0.028	2.491 ± 0.194	0.153 ± 0.017	0.198 ± 0.018	0.593 ± 0.021	0.024 ± 0.004	0.048 ± 0.006	0.179 ± 0.090

* *P* < 0.05, ** *P* < 0.01, *** *P* < 0.001, significant difference from the control group.

**Table 2:** The blood biochemical parameters of rats after 3 months of repeated administration and 2 weeks of recovery, respectively (mean ± SD, *n* = 12 for administration and *n* = 6 for recovery).

Parameters		ALT	AST	ALP	TBIL	TC	TG	TP	ALB	BUN	CRE	CK	GLU
12 weeks	Control	29.00 ± 5.59	121.09 ± 19.11	150.36 ± 52.87	0.23 ± 0.10	1.00 ± 0.17	0.84 ± 0.20	56.48 ± 7.46	25.87 ± 1.68	5.95 ± 2.03	69.71 ± 11.18	968.55 ± 373.69	13.86 ± 1.28
	P14 mg/kg	27.92 ± 8.34	116.75 ± 11.96	54.00 ± 12.38 ***	0.22 ± 0.07	1.18 ± 0.26	0.50 ± 0.09 ***	54.54 ± 2.01	23.34 ± 0.96 ***	3.26 ± 0.69 ***	66.13 ± 3.12	375.67 ± 110.47 ***	11.68 ± 2.47 *
	P28 mg/kg	26.25 ± 8.55	130.67 ± 38.00	46.25 ± 9.92 ***	0.30 ± 0.12	1.02 ± 0.42	0.86 ± 0.43	55.81 ± 3.25	24.12 ± 1.13 **	3.85 ± 0.67 **	66.58 ± 5.56	420.92 ± 210.98 ***	8.48 ± 2.24 ***
	P56 mg/kg	25.00 ± 4.61	115.92 ± 26.82	55.83 ± 14.86 ***	0.28 ± 0.11	1.01 ± 0.39	0.84 ± 0.40	54.99 ± 2.52	24.47 ± 1.46 *	3.21 ± 0.62 ***	67.50 ± 4.12	535.58 ± 209.83 **	11.67 ± 1.39 ***
	IP28 mg/kg	27.00 ± 5.95	109.83 ± 25.96	50.17 ± 9.19 ***	0.22 ± 0.08	1.27 ± 0.41	0.68 ± 0.19	55.71 ± 1.99	24.32 ± 1.25 *	3.59 ± 0.6 ***	66.39 ± 8.78	518.33 ± 246.45 **	10.39 ± 3.94 *
Recovery	Control	36.33 ± 5.92	97.33 ± 11.33	71.33 ± 30.03	0.23 ± 0.09	1.54 ± 0.28	0.61 ± 0.36	59.45 ± 2.70	26.27 ± 1.44	3.81 ± 0.22	68.20 ± 2.05	564.83 ± 166.87	12.53 ± 2.81
	P14 mg/kg	37.33 ± 3.67	108.17 ± 7.76	88.83 ± 29.55	0.18 ± 0.06	1.49 ± 0.15	1.31 ± 0.83	61.00 ± 3.48	27.41 ± 1.84	4.50 ± 0.47 **	68.58 ± 2.63	895.00 ± 342.46	13.56 ± 2.70
	P28 mg/kg	38.50 ± 4.23	104.50 ± 17.74	60.33 ± 7.94	0.23 ± 0.09	1.58 ± 0.25	0.62 ± 0.22	60.40 ± 0.85	26.72 ± 0.87	3.86 ± 0.51	69.09 ± 4.47	689.83 ± 206.08	13.83 ± 0.92
	P56 mg/kg	38.00 ± 4.60	114.33 ± 11.33 *	91.17 ± 29.26	0.21 ± 0.05	1.23 ± 0.18 *	0.55 ± 0.18	59.49 ± 1.84	26.25 ± 1.49	4.50 ± 0.49 *	67.44 ± 3.32	840.83 ± 442.98	13.61 ± 1.94
	IP28 mg/kg	34.40 ± 3.13	89.60 ± 7.89	60.80 ± 14.81	0.23 ± 0.07	1.49 ± 0.41	0.44 ± 0.13	59.56 ± 1.65	25.91 ± 1.47	3.62 ± 0.54	67.09 ± 3.65	814.40 ± 550.44	12.46 ± 2.13

* *P* < 0.05, ** *P* < 0.01, *** *P* < 0.001, significant difference from the control group.

**Table 3:** Hematological values of rats after 3 months of repeated administration and 2 weeks of recovery, respectively (mean ± SD, *n* = 12 for administration and *n* = 6 for recovery).

Parameters		WBC (10^9^/L)	RBC (10^12^/L)	HGB (g/L)	MCV (fl%)	MCH (pg)	MCHC (g/L)	PLT (10^10^/L)
12 weeks	Control	1.88 ± 1.14	4.34 ± 1.93	85.42 ± 37.08	54.67 ± 2.50	19.83 ± 0.73	362.67 ± 13.55	421.67 ± 233.20
	P14 mg/kg	4.07 ± 2.66 *	5.59 ± 1.47	111.08 ± 24.58	53.74 ± 2.90	20.08 ± 1.25	373.92 ± 12.84 *	650.58 ± 346.52
	P28 mg/kg	2.98 ± 4.77	3.96 ± 2.10	76.25 ± 41.05	53.55 ± 3.04	19.34 ± 1.01	361.67 ± 14.39	430.33 ± 425.39
	P56 mg/kg	2.72 ± 2.09	4.10 ± 2.36	72.00 ± 48.09	55.04 ± 4.31	16.84 ± 6.47	304.00 ± 112.82	515.83 ± 396.29
	IP28 mg/kg	3.53 ± 2.38 *	5.11 ± 1.94	38.58 ± 17.45 ***	54.10 ± 3.54	7.34 ± 2.60 ***	135.00 ± 45.26 ***	620.17 ± 423.04
Recovery	Control	3.02 ± 0.75	6.91 ± 0.51	138.67 ± 3.39	53.42 ± 2.61	20.15 ± 1.36	377.17 ± 7.73	959.83 ± 75.28
	P14 mg/kg	2.73 ± 0.87	6.44 ± 1.24	128.33 ± 26.17	53.42 ± 1.95	19.88 ± 0.65	372.00 ± 7.75	913.33 ± 193.37
	P28 mg/kg	2.35 ± 0.40	6.68 ± 1.19	130.52 ± 2.97	53.25 ± 1.47	19.55 ± 0.53	367.33 ± 9.79	970.17 ± 274.90
	P56 mg/kg	2.77 ± 0.59	7.08 ± 1.68	137.00 ± 31.10	53.00 ± 1.75	19.4 ± 50.85	366.67 ± 6.09 *	737.33 ± 313.05
	IP28 mg/kg	2.23 ± 0.99	7.98 ± 0.89 *	159.17 ± 18.05 *	53.22 ± 1.57	19.95 ± 0.63	375.00 ± 9.19	678.05 ± 414.28

* *P* < 0.05, ** *P* < 0.01, *** *P* < 0.001, significant difference from the control group.

**Table 4:** Validated parameters of partial least squares discriminate analysis (PLS-DA) models in pairwise comparisons.

PLS-DA Model	R^2^X (cum *^a^*)	R^2^Y (cum)	Q^2^ (cum)	Intercept of R^2^	Intercept of Q^2^	*P* value (CV-ANOVA)
TControl *vs*. TP14	0.542	0.983	0.762	0.973	0.281	0.044 *
TControl *vs*. TP28	0.402	0.989	0.813	0.947	−0.101	0.012 *
TControl *vs*. TP56	0.381	0.983	0.779	0.939	0.046	0.003 **
TControl *vs*. TIP28	0.445	0.995	0.814	0.988	0.211	0.066
RControl *vs*. RP14	0.304	0.965	0.284	0.923	0.138	0.952
RControl *vs*. RP28	0.374	0.923	0.310	0.930	0.186	0.872
RControl *vs*. RP56	0.389	0.997	0.932	0.961	0.032	0.393
RControl *vs*. RIP28	0.374	0.995	0.603	0.976	0.256	0.572

*^a^* cum: accumulation.

**Table 5:** The VIP, fold change and *P* values of 22 differential metabolites in drug treatment period.

Metabolites	Chemical Shifts	TControl *vs*. TP56			TControl *vs*. TP28			TControl *vs*.TP14			TControl *vs*.TIP28		
		VIP	FC	*P*	VIP	FC	*P*	VIP	FC	*P*	VIP	FC	*P*
Valine	0.968	1.958	0.665	0.007	2.091	0.531	0.009	1.071	0.825	0.458	0.880	0.905	0.843
Isoleucine	0.998	1.629	0.644	0.040	1.967	0.485	0.015	1.050	0.802	0.516	0.638	1.019	0.977
Isobutyrate	1.050	1.828	0.640	0.013	1.801	0.631	0.026	1.170	0.753	0.377	1.381	0.738	0.404
Ethanol	1.157	2.060	2.384	0.006	1.911	2.620	0.018	0.684	1.253	0.613	2.204	1.988	0.046
3-Hydroxybutyrate	1.187	1.604	0.361	0.040	1.543	0.287	0.073	1.057	0.644	0.470	1.534	0.332	0.291
Alanine	1.459	1.802	0.625	0.015	1.662	0.613	0.047	1.209	0.740	0.343	0.247	0.985	0.977
Acetone	2.218	2.011	0.366	0.007	2.137	0.271	0.007	1.132	0.602	0.421	1.782	0.310	0.148
Pyruvate	2.361	1.787	0.658	0.017	1.758	0.607	0.035	1.433	0.696	0.226	0.868	0.910	0.744
Glutamine	2.431	2.114	0.644	0.006	2.075	0.687	0.011	1.949	0.651	0.059	2.200	0.610	0.040
Citrate	2.527	1.945	0.474	0.007	1.999	0.447	0.013	1.067	0.838	0.623	1.050	0.774	0.574
PUFA	2.775	2.011	0.578	0.007	1.763	0.538	0.030	1.802	0.708	0.052	1.970	0.601	0.082
Dimethyl sulfone	3.14	1.876	1.555	0.011	1.862	1.814	0.020	1.604	1.272	0.379	2.302	1.881	0.026
Choline	3.187	1.799	0.582	0.016	1.987	0.538	0.013	0.989	1.066	0.809	1.148	1.173	0.611
Proline	3.318	1.718	1.594	0.026	1.389	1.550	0.126	1.774	1.573	0.252	2.161	2.076	0.050
Glycine	3.55	1.916	0.624	0.011	1.517	0.560	0.099	1.278	0.610	0.401	1.349	0.693	0.404
Creatine	3.919	2.096	0.532	0.006	2.048	0.471	0.012	1.694	0.459	0.158	1.753	0.561	0.159
Adenosine	6.045	1.621	0.532	0.040	1.141	0.566	0.342	1.329	0.492	0.302	2.030	0.454	0.069
Thymidine	6.259	1.838	0.296	0.012	1.489	0.354	0.104	1.595	0.361	0.120	1.652	0.366	0.211
Tyrosine	7.185	1.553	1.997	0.049	1.377	1.713	0.123	0.555	1.117	0.820	1.412	1.631	0.389
Phenylalanine	7.304	1.682	0.581	0.029	1.823	0.496	0.025	0.945	0.739	0.506	0.827	0.816	0.722
4-Hydroxybenzoate	7.822	1.759	0.500	0.024	1.741	0.407	0.041	1.195	0.484	0.395	1.610	0.549	0.262
Formate	8.441	1.819	0.382	0.029	1.590	0.236	0.072	0.842	0.634	0.578	1.581	2.223	0.251

**Table 6:** The VIP, fold change and *P* values of 22 differential metabolites in recovery period.

Metabolites	Chemical Shifts	RControl *vs*. RP56			RControl *vs*. TP28			RControl *vs*. RP14			RControl *vs*. RIP28		
		VIP	FC	*P*	VIP	FC	*P*	VIP	FC	*P*	VIP	FC	*P*
Valine	0.968	1.229	1.322	0.537	0.753	1.048	0.926	1.233	0.923	0.918	1.490	1.228	0.878
Isoleucine	0.998	1.209	1.264	0.546	0.651	1.030	0.959	1.642	0.876	0.840	1.666	1.360	0.797
Isobutyrate	1.050	0.741	0.864	0.687	1.388	0.752	0.848	1.159	0.853	0.937	1.602	0.779	0.723
Ethanol	1.157	0.343	1.017	0.975	0.542	0.908	0.929	0.734	1.183	0.999	0.700	0.900	0.894
3-Hydroxybutyrate	1.187	0.861	1.434	0.656	0.853	0.966	0.986	0.267	1.009	0.999	0.665	1.044	0.981
Alanine	1.459	0.018	1.003	0.995	1.059	0.836	0.848	2.120	0.838	0.663	0.407	0.989	0.983
Acetone	2.218	0.185	0.950	0.941	0.673	0.981	0.993	0.681	1.157	0.999	0.251	0.985	0.986
Pyruvate	2.361	1.052	1.176	0.603	0.928	1.113	0.867	0.428	1.075	0.999	0.970	1.195	0.884
Glutamine	2.431	1.679	0.780	0.416	2.011	0.683	0.848	1.816	0.828	0.763	1.968	0.732	0.575
Citrate	2.527	0.200	1.041	0.950	1.013	0.871	0.867	1.949	0.757	0.692	1.494	1.346	0.878
PUFA	2.775	0.548	1.081	0.852	0.128	0.981	0.977	0.778	1.085	0.999	0.324	1.015	0.984
Dimethyl sulfone	3.140	1.141	0.717	0.562	1.340	0.776	0.848	1.110	0.834	0.938	1.831	0.670	0.636
Choline	3.187	1.790	1.535	0.405	0.671	1.087	0.883	0.132	0.990	0.999	1.993	1.473	0.575
Proline	3.318	0.732	0.790	0.691	0.947	0.810	0.857	1.504	0.649	0.904	1.687	0.680	0.693
Glycine	3.550	1.603	0.680	0.422	1.361	0.776	0.848	1.978	0.799	0.687	1.309	0.836	0.852
Creatine	3.919	1.401	0.772	0.481	1.617	0.746	0.848	1.219	0.881	0.918	1.213	0.786	0.878
Adenosine	6.045	0.272	0.923	0.916	1.071	0.712	0.848	1.472	0.684	0.904	1.269	0.674	0.876
Thymidine	6.259	0.888	1.265	0.653	0.815	0.891	0.874	1.138	0.811	0.938	1.159	0.841	0.878
Tyrosine	7.185	1.364	0.525	0.496	0.875	0.799	0.848	0.427	1.047	0.999	1.593	0.676	0.737
Phenylalanine	7.304	1.273	1.262	0.524	0.899	0.910	0.848	0.186	1.009	0.999	0.684	1.068	0.929
4-Hydroxybenzoate	7.822	0.474	0.880	0.823	0.472	0.915	0.932	0.262	1.046	0.999	1.568	0.719	0.742
Formate	8.441	1.600	2.000	0.426	1.194	1.474	0.848	0.868	1.099	0.999	2.145	1.797	0.432
